# Herbivore-Alga Interaction Strength Influences Spatial Heterogeneity in a Kelp-Dominated Intertidal Community

**DOI:** 10.1371/journal.pone.0137287

**Published:** 2015-09-11

**Authors:** Moisés A. Aguilera, Nelson Valdivia, Bernardo R. Broitman

**Affiliations:** 1 Centro de Estudios Avanzados en Zonas Áridas (CEAZA), Universidad Católica del Norte, Larrondo 1281, Coquimbo, Chile; 2 Instituto de Ciencias Marinas y Limnológicas, Facultad de Ciencias, Universidad Austral de Chile, Campus Isla Teja s/n,Valdivia, Chile; The University of Sydney, AUSTRALIA

## Abstract

There is a general consensus that marine herbivores can affect algal species composition and abundance, but little empirical work exists on the role of herbivores as modifiers of the spatial structure of resource assemblages. Here, we test the consumption/bulldozing effects of the molluscan grazer *Enoplochiton niger* and its influence on the spatial structure of a low intertidal community dominated by the bull kelp *Durvillaea antarctica* and the kelp *Lessonia spicata*. Through field experiments conducted at a rocky intertidal shore in north-central Chile (~30°-32°S), the edge of the grazer and algae geographic distributions, we estimated the strength and variability of consumptive effects of the grazer on different functional group of algae. We also used data from abundance field surveys to evaluate spatial co-occurrence patterns of the study species. Exclusion-enclosure experiments showed that *E*. *niger* maintained primary space available by preventing algal colonization, even of large brown algae species. The grazing activity of *E*. *niger* also reduced spatial heterogeneity of the ephemeral algal species, increasing bare space availability and variability through time in similar ways to those observed for the collective effect with other grazers. Overall, our result suggests that *E*. *niger* can be considered an important modifier of the spatial structure of the large brown algae-dominated community. Effects of *E*. *niger* on resource variability seem to be directly related to its foraging patterns, large body size, and population densities, which are all relevant factors for management and conservation of the large brown algae community. Our study thus highlights the importance of considering functional roles and identity of generalist consumers on spatial structure of the entire landscape.

## Introduction

Determining the variation in the magnitude and direction of species interactions and its ecological consequences through field experiments is critical to understand the functioning of diverse consumer assemblages, because the spatial structure of these ecological processes determines the scenario for species coexistence [1–8]. In aquatic ecosystems, benthic consumers significantly influence lower trophic levels’ composition, abundance and distribution, and thus the web of trophic interactions [1,4,9,10]. Consumption by herbivores can greatly modify the spatial structure of their habitat via consumption or bulldozing effects on dominant or habitat-forming species [11–15]. Spatial effects of consumer impacts are especially relevant in human-disturbed ecosystems, where keystone species with large body sizes are removed and functional compensation is supressed by species-specific overexploitation [16–19]. Hence, identifying the specific roles of consumer species and their potential to modify the spatial structure of their habitats is of great interest to guide conservation and management strategies across different spatial scales.

In marine intertidal communities, benthic grazers can play important roles determining algal assemblage composition through both negative and positive consumption effects on numerically and functionally dominant species [6,10,20,21]. Evidence suggests that specific grazing effects are dependent on the diversity of roles of the resident guild, and grazer-specific traits can have major effects on the spatial structure of their habitat and resources [18,22–27]. In general, both the magnitude and direction of grazer effects depend on species characteristics like body size, population densities, foraging mode, and algal life stages and morphology (see ref. [10] for review) and interactions with the environment are also important (e.g. [28,29]). Molluscan grazers that scrape the rocky substrata can reduce algal biomass, consuming spores and juveniles of macroalgae [20,30–32]. This grazing strategy can alter early community succession and the composition of algal assemblages at different spatial scales. Notwithstanding, spatial variance in grazing effects hinges on the distribution of individuals when foraging and intrinsic individual variation, which are a direct function of population densities and resource spatial distribution [22,23,25,28,33,34]. For example, grazers with gregarious foraging patterns can create patchy distribution patterns of algal species, prescribing scales of spatial heterogeneity of the resident algal assemblage [11,23,25,35]. On one hand, herbivores of large body size have higher resource requirements and forage over broad areas, potentially homogenizing resource distribution [13]. On the other hand, herbivores of large body size could have large mean consumptive effects but this can be spatially variable thus enhancing resource spatial heterogeneity (see refs. [22,25,36,37] for consumer-resource models).

Large brown algae (i.e. kelps of fucoids) represent nutritional resources and habitat for diverse and abundant associated communities [38,39]. In these habitats, benthic grazers can significantly influence algal species abundance and production, potentially modifying the spatial distribution of dominant species [4,38–40]. Experimental studies have shown that benthic grazers can have strong *per capita* effects on the settlement of dominant kelp or fucoid species, influencing assemblage composition in these systems [2,4,10,41,42]. Thus, distribution and density patterns of a diverse benthic grazer guild can be critical for kelp forest community composition and recovery following disturbances [4,35,43]. The effects of grazing activity on the structure of kelp communities depend on grazer body sizes and kelp abundance: while intermediate-size grazers can dramatically modify kelp forest structure when kelps show intermediate values of abundances, large grazers are important when kelps show high abundances [1,4,44]. For example, considering population-level estimates of grazer impacts from the sea urchin *Strongylocentrotus purpuratus* it was possible to predict a shift from a species-rich subtidal kelp forest to sea urchin barrens after the reduction of top predators allowed for higher grazer densities [2,40]. This pattern suggests that the disproportionate mean effects and densities of some grazers can correspond with a homogenization of the spatial structure of the resource community (i.e. continuous distribution of calcareous algae or bare rock, [40]). Grazer-driven homogenization of resource distribution can potentially reduce kelp forest resilience affecting recovery rates and persistence of local populations (e.g. [1,40,43]). Large brown algae are important habitat-forming species, so changes of the spatial str0075cture of adult stands may propagate to the local community [38].

**Fig 1:**
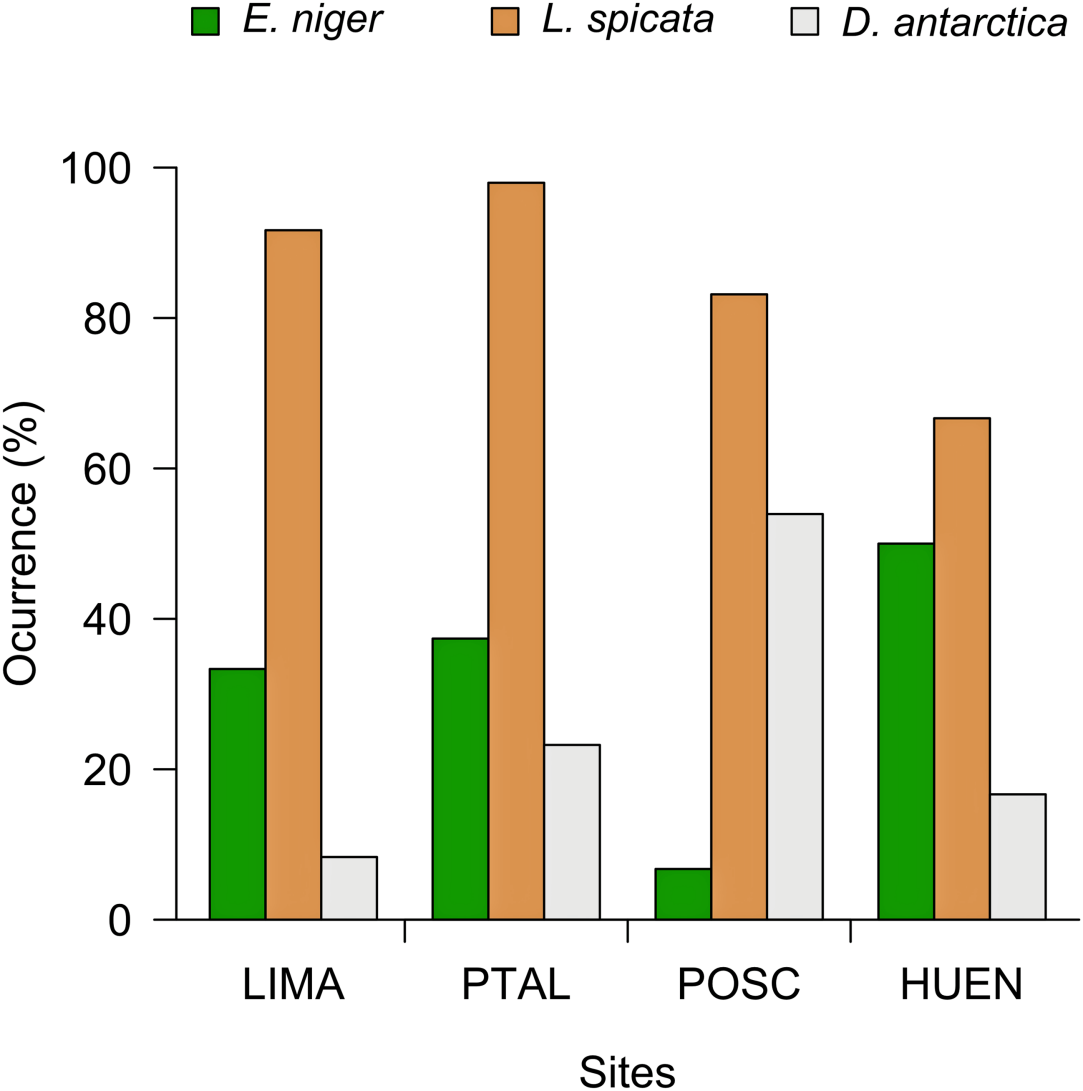
Percentage of plots where the grazer *E*. *niger* and the brown algae *L*. *spicata* and *D*. *antarctica* were recorded at four sites present in northern Chile. Sites: LIMA: Limarí (30°45’S-71°42’W); PTAL: Punta Talca (30°55’S-71°40’W); POSC: Puerto Oscuro (31°25’S-71°36’W); HUEN: Huentelauquén (31°38’S-71°33’W).

Along the coast of northern-central Chile, intertidal kelps sustain different species assemblages and are focus of intense harvesting [39,45]. A broad transitional zone located between 30°S-41°S includes a 200-km wide section on the northern edge of this area, between 30°S-32°S, that is the polar or equatorial edge of the geographic range of several intertidal species [46–48]. Two species of brown algae, the bull kelp *Durvillaea antarctica* and the kelp *Lessonia spicata*, form dense mixed stands in the low intertidal zone, and reach their equatorward limit of distribution at ~30°S. Across the same region, the polar limit of large (~10 cm) and abundant molluscan grazer *Enoplochiton niger* [49] occurs at ~32°S; this species inhabits in sympatry with the two kelps at the transition zone [46,50–52] (see Fig 1). Previous observations suggest that given large densities and body size of the chiton *E*. *niger* (compared with other species of the assemblage, [49], this species could have large effects on the algae assemblage structure especially affecting recruitment rates of large brown algae stands [53]. Dense populations of this grazer observed in northern Chile (beyond 30°S), seem to be a critical factor maintaining high bare rock cover interspersed with patches of morphologically different algae species. No studies have been conducted exploring the role of this chiton in structuring algae assemblage in kelp-dominated system.

Here we examine the specific role of the chiton *E*. *niger* and the spatial variability of its consumptive impacts on intertidal kelp communities. Using a mixture of field observational surveys and manipulative experiments conducted in northern Chile, we examined the effects of grazing by *E*. *niger* on the spatial structure of kelp stands of the low intertidal zone. We hypothesized that (a) given the large body size and densities of *E*. *niger*, this species will have large mean *per capita* and population-level consumption/bulldozing effects through direct grazing and/or mechanically removing the early life stages of different algae species. Thus this species could reduce the spatial variability in algal abundance and bare rock cover, i.e. a “homogenising spatial effect”. Alternatively, (b) given that *E*. *niger* could have a random or uniform individual spatial distribution while foraging, this species could determine large variance on algal abundance i.e. a “heterogeneous spatial effect”. We tested these hypotheses on functionally distinct species of algae, such as opportunistic/ephemeral foliose algae (ulvoids and *Pyropia* sp.) and large brown algae species (*Lessonia* and *Durvillaea*), and on rates of production and maintenance of bare rock which is directly correlated with algae consumption and bulldozing effect of the grazer species.

## Materials and Methods

### Ethics Statement

All invertebrate manipulation in the field was conducted according to relevant national and international guidelines. In addition, the study sites are not privately owned, so that no permits for access were needed.

### Focal Species and Study Site

The low intertidal zone in the study site was characterized by dominance of the large brown algae *Lessonia spicata* and *Durvillaea antarctica* [39,54]. Opportunistic algae are characterized by *Ulva rigida*, *U*. *compressa*, *Hincksia michelliae* and *Pyropia orbicularis* and *Porphyra* spp., which are abundant from high to low-intertidal levels. Patches of the crusts *Hildenbrandia lecannellieri*, *Ralfsia* sp., *Corallina chilensis*, and *Lithothamnion* sp. are frequently interspersed with opportunistic forms [53]. The kelp *L*. *spicata* (corresponding to southern species of the *L*. *nigrescens* complex, see ref.[52]) and the bull kelp *D*. *antarctica* form a dense canopy in low intertidal levels, supporting a diverse invertebrate assemblage [54,55]. The herbivore assemblage of the low intertidal zone hosts several grazer species, including the large chitons *E*. *niger*, *Acanthopleura echinata*, and *Chiton granosus*, the keyhole limpet *Fissurella limbata*, and fish that venture onto rocky intertidal platforms. Both turban snails (*Tegula* spp.) and the sea urchins *Tetrapygus niger* and *Loxechinus albus* are commonly absent in wave exposed platforms, but form dense patches in low intertidal pools in more protected areas.

Holdfasts of *L*. *spicata* similar to other kelps have been recognized as ‘micro-ecosystems’ in which diverse invertebrate species live [39,56], constituting a habitat-forming species [54]. The bull kelp recruits massively as single plants for around two to three seasons, while *L*. *spicata* have year-round recruitment (previously considered as *L*. *nigrescens* in [33,41,44]). Both species are intensely harvested in Chile, which involve fronds cut or removal of entire plant (e.g. [45]). *E*. *niger* is among the largest molluscan herbivores of the assemblage present north of the range overlap (mean ± Standard Error of the Mean of maximum body length: 10.9 ± 0.13 cm, and see [49]), reaching high densities and frequencies of occurrence at the northern limit of the overlap zone (6.65 ± 0.59 ind. m^-2^, Fig 1B and 1C). It is a generalist grazer capable of consuming spores and juvenils of macroalgae including *Lessonia* spp. [57,58]. However, no studies have been conducted to determine the strength of its consumptive effects on the intertidal community. According to its radular scraping capabilities, this species can be considered an efficient grazer, capable of removing macroalgal spores and plantlets at very high rates [59]. Basic ecological information (species richness of preys, grazer size structure, abundance and diet: [48,50,58]) suggests this grazer might have large *per capita* and population-level consumptive effects on kelp-dominated algal communities, potentially affecting algal spatial distribution and recruitment at local scales [53]

### Spatial distribution patterns of *E*. *niger* and algae

To determine the spatial distribution of grazer and algal species (i.e., if were randomly, aggregated, or uniformly distributed over rocky platforms), and to quantify the level of spatial association (i.e. spatial correlation at lag 1) between the herbivore-brown algae pair, we recorded *E*. *niger* density and algal cover at different seasons in the locality of Punta Talca (Fig 1). We recorded the percentage cover of algae using 17 30×30 cm contiguous quadrat (81 uniformly spaced intersection points) positioned along transects parallel to the shoreline on the low intertidal level. Densities of *E*. *niger*, *D*. *antarctica*, and *L*. *spicata* recruits and adult plants were estimated using 12 50 × 50 cm contiguous quadrats placed in low-shore alongshore transects. The samplings were conducted at two intertidal platforms, about 15 m length. All sampling protocols were conducted between October 2010 and July 2011.

### Herbivore-algae interaction strength: field experiments

#### *E*. *niger* effects on algal colonization (Experiment 1).

The bull kelp *D*. *antarctica* and *L*. *spicata* have leathery morphologies [60] and, in comparison with spores, plantlets, and young plants, adult plants are seldom grazed or browsed by benthic herbivores [10,61]. Therefore, population control of the brown algae species by *E*. *niger* is expected through direct consumption of spores and plantlets [58], which could scale up to the entire algal assemblage structure (see [53]). A field experiment, conducted in the low intertidal in Punta Talca, was designed to determine the consumptive effect of *E*. *niger* on recruitment of *D*. *antarctica* and *L*. *spicata* and different morpho-functional group of algae (according to [60]) and on bare substrata maintenance (see S1 File). In this experiment, each experimental unit consisted of a 35 × 35 cm area, which we previously observed allows to adult *E*. *niger* individuals ample movement during foraging. All plots were scraped clean with drill-mounted brushes and manual chisels, thus removing all organisms including encrusting algal fragments. This procedure resets the community to early successional stages (100% bare rock cover). Four experimental units were randomly assigned to each of the following three treatments: a) Enclosure of one individual of *E*. *niger* (10.7 ± 0.13 cm body length), according to natural densities of this chiton in the range overlap in low intertidal levels ([50] and see Results), b) Exclusion of benthic grazers, where all herbivores were removed from the plots, and c) Control ‘open access’ areas (i.e. with no fences) where all herbivores were allowed to enter and graze (**Figure A in** S1 File). Thus, this experimental design allowed us to examine the overall effect of the grazer presence/absence on algae abundance (i.e. percent cover differences between treatments) at different times of the community succession. We enclosed/excluded benthic grazers using stainless steel fences (7 cm high, 7 mm mesh opening) 35 × 35 cm area, fastened to the rock with stainless-steel bolts, proved as an effective field experimental procedure to reduce benthic grazer migration in this (see ‘preliminary experiments’ in S1 File) and other [24] studies. A preliminary study showed that using partial fences as a procedural control treatment was not adequate for this experiment (see **Figure A in** S1 File). Since partial fences were frequently damaged by waves, they can cause undesirable effects on algae colonization and were thus not further considered in the final experimental design. Additionally, our preliminary studies (see S1 File) and previous studies suggest that different exclusion methods (e.g. plastic brush and copper paint) seems not to alter significantly algae recolonization onto emergent substrate in rocky intertidal habitats compared with open areas where the large bare rock presence is attributed to the grazing of the diverse intertidal herbivore assemblage [62–64]. Thus we expected that the effect of fences, if were any, on altering algal cover was minimal.

From December 2009 to May 2010 we monitored monthly the percentage cover of all macrobenthic (> 3 cm) sessile organisms in each experimental area using 30 × 30 cm quadrats (81 uniformly spaced intersection points). At each sampling date, the whole benthic community in each plot was photographed with a high-resolution digital camera and percentage cover estimations were conducted in the laboratory. The percentage cover of bare space inside experimental areas was considered as a direct measure of foraging rate of the grazer species compared with exclusions (grazer-free areas). In order to obtain information of grazing intensity in the experimental “open access areas” in the experiments, and to follow algal settlement, we estimated every two months the density of herbivores and both adult and juvenile *L*. *spicata* and bull kelps present on the experimental platform (see **Figure A in** S1 File).

#### Grazing effects of *E*. *niger* on plantlets of *D*. *antarctica* (Experiment 2).

Because the bull kelp did not recruit within experimental plots in our first series of experiments (see Result section) we examined the consumptive effect of *E*. *niger* on *D*. *antarctica* small plantlets (< 5 cm) by means of a second independent experiment in the low intertidal zone. In this experiment, we transplanted small plantlets of *D*. *antarctica* (0.5–2.0 cm frond length) inside experimental areas, following previous experimental approaches involving transplant of small plantlets (see [61]). *D*. *antarctica* plantlets were carefully scraped off from the substratum, weighted, frond length measured, and then glued with polyacrylamide glue directly onto acrylic plates (3.0 x 3.0 x 0.1 cm). Two small plantlets were attached to each plate, one in the center and the other in the edge of the plate in order to control for potential effects of the position on the plate. Plates were affixed to the rock with a flat-head screw flush to the plate surface and placed in the middle of each experimental plot. Each plate with two small plantlets was randomly assigned to the different replicate of the following four treatments (n = 4): a) *E*. *niger* enclosure at natural densities, b) benthic grazer exclusion areas, c) control (open access) and d) a procedural control (partial fences) to control for potential artefact of the exclusion method (fences) on plantlets survival and growth. Procedural control consisting of partial fences was easily damaged by waves in preliminary studies, affecting rate of spore colonization especially at the edges of the experimental plots (see S1 File). Given that in this second experiment we examine effects of grazers on plantlets growth and survival, this procedural control was consider appropriate to examine the potential effect of fences on these variables. Despite the rate of damaged caused by waves on partial fences, plantlets growth and survival were not different on this treatment compared with control (open) plots (**Table B in** S1 File). Thus, this experimental design allowed us to determine the direct consumptive effect and potential for bulldozing effects (mechanical removal) on plantlets frond length and biomass, independently of their effects on spore settlement and re-colonization, which were evaluated in experiment 1. Every 25 days from November 2013 to January 2014, we removed the plates, measured frond length and weight of each *D*. *antarctica* plantlets, and then deployed new plates with new plantlets inside the experimental plots. This procedure allowed us to estimate changes in biomass and frond length of plantlets.

### Data Analysis

The small-scale spatial structures (cm to meters) of grazer and algal abundance data were analyzed using spatial correlograms based on Moran’s *I* [65]. We determined significance (α = 0.05) bootstrapping our observations [66]. The significance at each lag was calculated with the distribution of autocorrelation coefficients obtained by randomly re-sampling the data set and recalculating the coefficients 1000 times. A global autocorrelation test was conducted by checking whether each lag contained at least one significant correlation after probabilities were adjusted using a Bonferroni correction for multiple test (α’ = 0.05/number of distance classes [65]).

Interspecific spatial correlations (r) of *E*. *niger* and large brown algae densities, and opportunistic and crustose forms cover at small-scales (cm to meters) measured with the contiguous quadrats, were estimated through a t-tests. For this analysis degrees of freedom were corrected based on the degree of spatial autocorrelation of the sampling data (i.e. densities and cover recorded in contiguous quadrats). For this correction, we used Moran’s *I* to estimate the spatial autocorrelation between data sets (quadrats at the same distance classes). Correlations at the study site (Punta Talca) were calculated on averaged abundance of both adult and juvenile *L*. *spicata* and *D*. *antarctica* individuals separately for summer (January to March) and winter (June to August) monthly surveys.

For experiment 1, percentage cover of ephemeral algae—i.e. pooled (sampling dates) percentage cover of ulvoids, *Pyropia* sp., and *Hincksia* sp.—and bare rock were analyzed using one-way repeated measures analysis of variance (RM-ANOVA) with time as within-subject factor and treatment as between-subject factor. Homogeneity of variance was graphically explored by means of residuals-vs.-fits and normal Q-Q plots. All data were thus log-transformed to improve variance homogeneity. The Hyund-Feldt correction was used to adjust degrees of freedom when data did not meet sphericity assumptions for univariate tests [67]. In the case of significant effects among treatment differences (between subjects) for experiment 1 and 2 we used the following planned contrasts: 1) to evaluate effects of all herbivores (total herbivory) we compared the control versus exclusion, 2) to evaluate the effects of enclosed *E*. *niger* versus other herbivores we compared the enclosure versus control, and 3) to evaluate the effect of *E*. *niger* in absence of other herbivores we compared enclosure versus exclusions. Dunn-Šidák correction was used to adjust significance levels for the multiple contrasts performed (see **Table A in** S1 File).

For the experiment 2, averaged changes, over three repetitions, in frond length and biomass of *D*. *antarctica* plantlets were analyzed considering the position of each plant attached to acrylic plates. Differences among treatments were tested with a split-plot ANOVA, considering the ‘position’ of plantlets inside acrylic plates (i.e. mid and outer) as a fixed and crossed factor, and plot as a random factor nested in treatment (whole-plot) (**Table B in** S1 File).

In order to determine the direction and magnitude of the herbivore effects on algae, and to provide more comparable information on interaction strength [68,69]), we estimated per capita interaction strength in the field experiments. Within plots, colonization of ephemeral algae started a few days after rock clearance and reached an established stage after 18–20 weeks. Benthic grazers mainly affect early life stages of algae, such as plantlets or recently settled spores. Hence, we estimated consumptive effects of natural densities of *E*. *niger* on algae and bare rock production considering their average cover pooled on all dates for the first twenty weeks of the study which corresponds well with the colonization phase of early successional algae species in the region [35,45]. In addition, we estimated the effect of *E*. *niger* on change of frond length and biomass of *D*. *antarctica* over three repetitions conducted in the experiment 2.

To quantify the interaction strength (per capita effects) considering natural densities of the grazer species, we used the “Dynamic Index” (DI), which is especially recommended for trophic interactions where resources exhibit positive exponential growth [69] as during early succession. The index was calculated as:

$$DI=\frac{\text{ln}\left[\frac{Cov_{EN}}{Cov_{EX}}\right]}{N\times t}$$

where Cov_EN_ is the mean specific algal cover in the herbivore enclosures, Cov_EX_ is the mean algal cover in the grazer exclusions, **N** is the density of herbivores in the experimental plots and **t** is the experiment duration, in this case in days. We also estimated population effects [3] of herbivores computing DI × natural density of the herbivore. Population-level effects allowed us to evaluate the potential impact of the grazer species on each algal species. An average population effect equal or >1 (100% of plants removed by herbivores) indicates either total prevention of algal recruitment or production of 1m^2^ bare rock per day. Confidence intervals (95%) for effects estimates were obtained through a bootstrapping procedure [66].

In order to quantify the effects of grazers on spatial variability (i.e. if effects increase or decrease spatial heterogeneity), we used the “effect size” metric following [25]:

$$ES=\text{ln}\left[\frac{\sigma^{2}{}_{+G}}{\sigma^{2}{}_{-G}}\right]$$

Where **σ** ^**2**^_**+G**_ correspond to the variance among replicates in presence of grazers (enclosure, control) in a particular date, and **σ** ^**2**^_**-G**_ is the variance in absence of grazers (exclusion). In order to examine changes in effect size through time, estimates of effect size were calculated for each sampling date and then averaged across the time span of the experiments. Confidence intervals (95%) for averaged effects estimates were obtained through a bootstrapping procedure as before. All analyses were conducted using the R environment version 3.1.0 [70].

## Results

### Spatial distribution patterns

**Fig 2:**
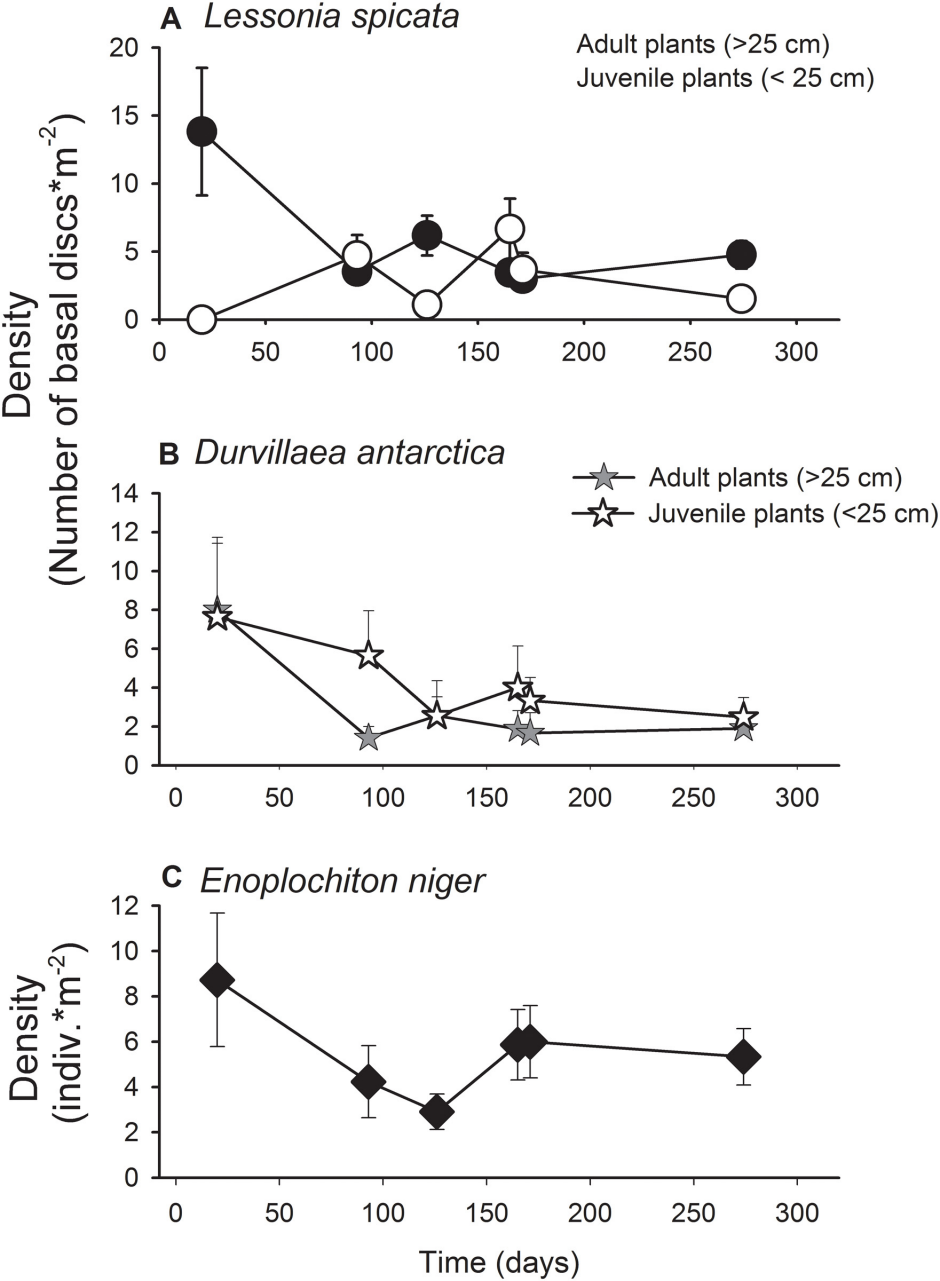
Average density (± SEM) of juvenile (small recruits from 3.0 to 25 cm frond length) and adult plants (longer than 25–30 cm) of (a) *Lessonia spicata* (b), *Durvillaea antarctica* and of the grazer (c) *Enoplochiton niger*, recorded through the study in the experimental platform (un-manipulated areas) at Punta Talca.

Small-scale spatial surveys (cm to meters) showed that adult *L*. *spicata* (>25 cm) were abundant at the beginning of the experiments, but then dropped to densities similar to those found for juvenile plants (Fig 2A). In the case of the bull kelp, adult and juvenile (<25 cm) individuals showed variable densities through the course of the study, with juvenile plants being more abundant during late summer, (i.e. 80 days from the beginning of the experiments Fig 2B). Densities of both adult and juvenile plants averaged across all dates were 2.9 ± 4.27 and 4.27 ± 0.82 plants m^-2^, respectively (Fig 2B). *E*. *niger* reached a density of 5.51 ± 0.79 ind. m^2^ at Punta Talca when averaging across all dates. This chiton was one of the most abundant grazer species present in the low intertidal zone of the study site, but showed large temporal variation in abundance (Fig 2C).

Intra-specific spatial structure at small scales (cm to meters) showed that *E*. *niger* was randomly distributed at the study site during summer and winter seasons (Table 1). For *D*. *antarctica*, distributional patterns of adults showed aggregated patterns during summer (50–100 cm) and random patterns during winter (Table 1a). Conversely, spatial distribution of juvenile *D*. *antarctica* individuals was random during summer and aggregated (50–142 cm) during winter (Table 1a). For adult and juvenile *L*. *spicata*, we found significant Moran’s I values at small spatial scales (50–150 cm) both during winter and summer indicating a patchy structure. Calcareous and non-calcareous crusts showed spatial structure with significant Moran’s I at the small scale during summer and negative but non-significant during winter (Table 1a). Ephemeral algae showed a random pattern of distribution during both summer and winter (Table 1a).

**Table 1:** Summary of Moran´s I autocorrelation at lag 1 (0–120 cm) (a) of *E*. *niger*, juvenile and adult individuals of *D*. *antarctica* and *L*. *spicata*, ephemeral and crustose algae during summer and winter and (b) Pearson spatial correlation (r) between densities of *E*. *niger* and algae functional groups recorded in the experimental platform. Modified t-tests were performed to determine significant differences in the herbivore-alga spatial correlation. Degrees of freedom and P-values were adjusted by presence of spatial autocorrelation in the data set (Dutilleul`s correction). Significance is indicated as P < 0.05*, P < 0.01** after random permutation test (1000 permutations).

**a)**		Summer	Winter
*Enoplochiton niger*		-0.068	0.150
		0.680	0.358
*Durvillaea antarctica*	Juvenile	0.285	-0.085
		0.039*	0.623
	Adult	0.042	0.689
		0.836	0.001**
*Lessonia spicata*	Juvenile	0.683	0.289
		0.001**	0.010*
	Adult	0.279	0.248
		0.010*	0.025*
Ephemerals		-0.008	-0.115
		0.823	0.650
Crusts		0.799	-0.206
		0.001**	0.380
**b)** *Enoplochiton niger*		Summer	Winter
*Durvillaea antarctica*	Juvenile	-0.247	-0.100
		0.336	0.681
	Adult	-0.148	0.388
		0.552	0.176
*Lessonia spicata*	Juvenile	-0.324	0.109
		0.185	0.632
	Adult	0.227	0.152
		0.396	0.043*
Ephemerals		0.327	-0.123
		0.274	0.627
Crusts		0.446	0.441
		0.103	0.161

Abundance of *E*. *niger* in the low intertidal zone was not correlated at the quadrat scale with density of adult plants of *D*. *antarctica* in winter nor in summer (Table 1b). Similarly, no spatial correlation was detected for *E*. *niger* with juvenile individuals of the bull kelp during summer or winter surveys (Table 1b). Significant positive spatial correlation was found for *E*. *niger* with adult *L*. *spicata* during winter (Table 1b), but no relationship was observed between *E*. *niger* and juvenile *L*. *spicata* (Table 1b). Similarly, no significant spatial relationship was observed between the abundance of *E*. *niger* and ephemerals nor crustose algae in any season considered (Table 1b).

### Herbivore-algae spatial variation in interaction strength

#### Grazer effects on species colonization and functional groups (Experiment 1)

**Fig 3:**
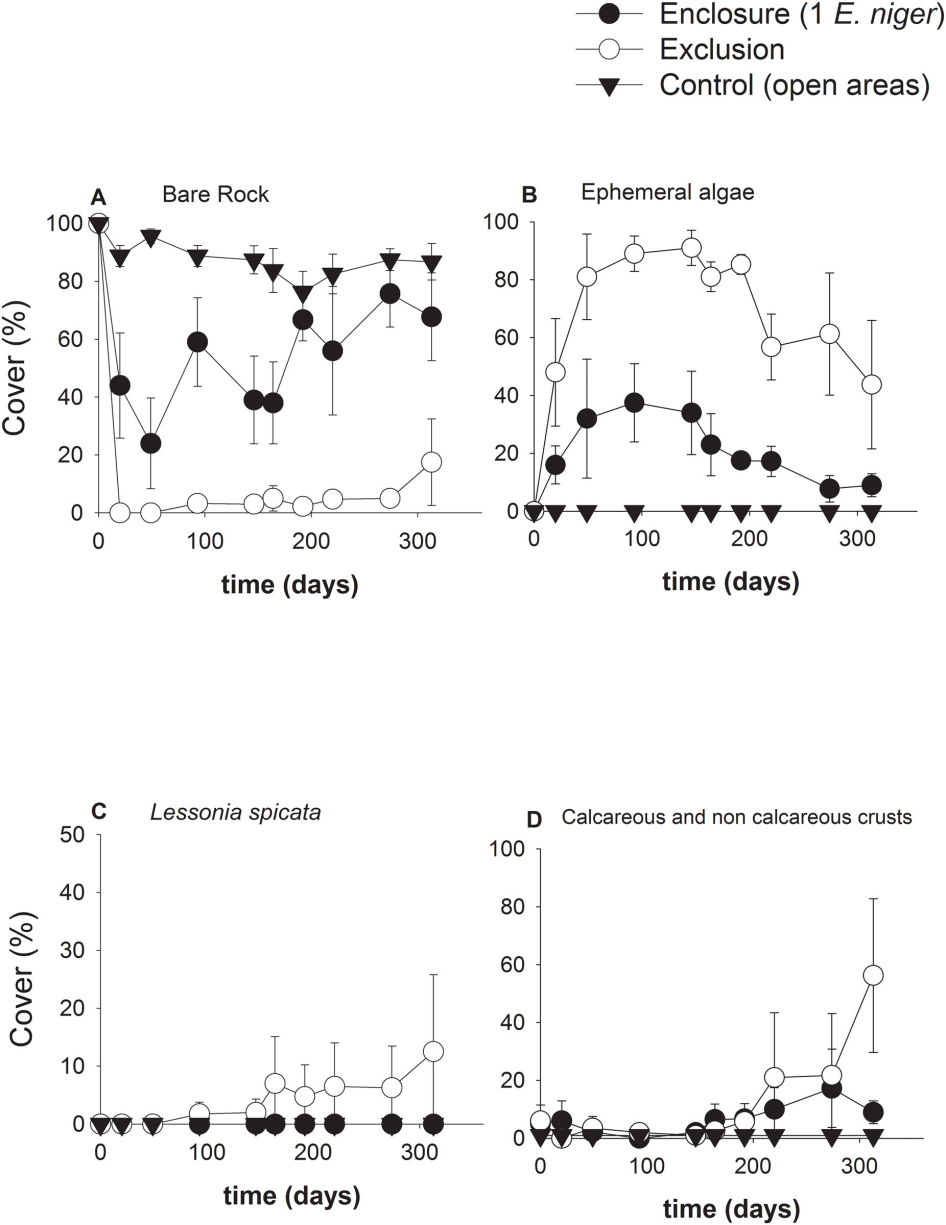
Monthly mean cover (±SE) of (a) bare rock; (b) ephemeral/opportunistic algae (i.e. *Ulva rigida*, *U*. *compressa*, *Pyropia* sp, *Hincskia* sp. *Polysiphonia* sp, ceramials) (c) *Lessonia spicata* and (d) crustose algae (i.e. encrusting coralline algae, *Hildenbrandia lecanelleri*) found inside experimental plots at Punta Talca.

We found differences in species abundances among treatments in our field experiments (see **Table A in** S1 File, Fig 3). *E*. *niger* maintained a high availability of bare substrate inside plots (Fig 3A), significantly reducing the abundance of ephemeral algae when compared with grazer exclusion areas (Fig 3B, see **Table A in** S1 File). Control areas, to which all benthic grazer had access, maintained high availability of primary substrate (Fig 3A), differing significantly with both enclosure and exclusion treatments (see planned contrasts in **Table A in** S1 File). In exclusion areas, ephemeral algae i.e. ulvoids, *Pyropia* sp. and *Hincksia* sp., dominated the experimental community until winter 2011 (~300 days from the start of experiments, see Fig 3C). The kelp *L*. *spicata* was able to recruit into exclusion plots during the experiment and remained below 20% throughout the study (Fig 3C). Inside enclosures of *E*. *niger*, or in presence of other herbivores (about 4 indiv. per plot) in control areas, neither *L*. *spicata* nor bull kelp were observed until the end of the experiment (~350 days). Crustose algae forms (i.e. encrusting coralline and non-calcareous algae *Hildenbrandia lecanelleri*) were observed inside enclosure and exclusion areas towards the end of the experiment when they increased in abundance in exclusion areas (Fig 3D).

#### Grazer effects on length and biomass of plantlets (Experiment 2)

In this second experiment, plantlets grew longer—albeit not significantly—in chiton exclusion than enclosures and controls (open areas and procedural control, respectively) areas. Neither treatments nor position of plantlets showed significant effects on plantlet growth (see **Table B in** S1 File). The same pattern was observed for plantlet biomass, in which differences were not significant (see **Table B in** S1 File).

#### Strength and variability of grazer effects

**Fig 4:**
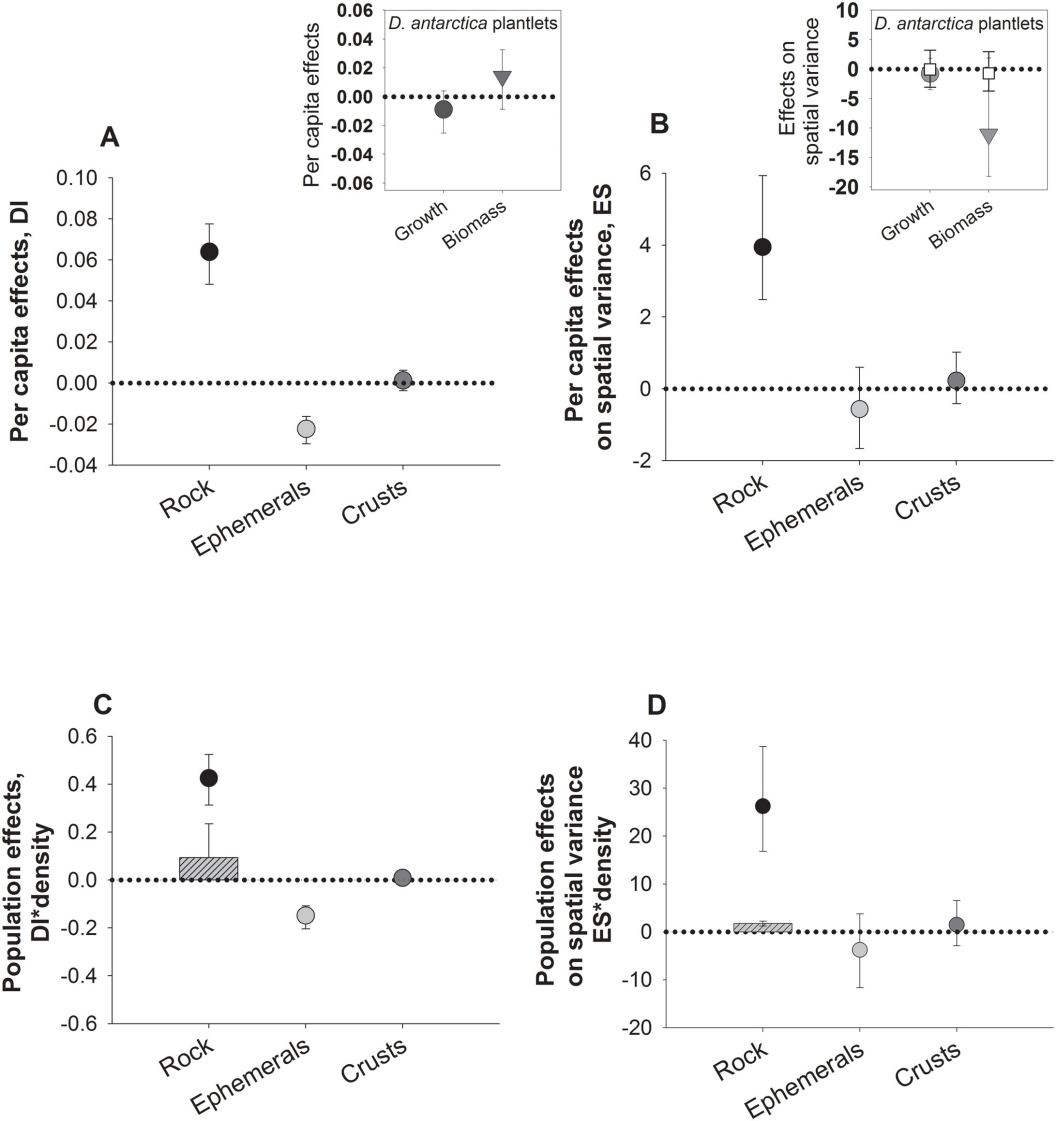
Strength of the interaction between the grazer *E*. *niger* and algae measured as the grazer capacity to influence the recruitment of algae and bare rock production during early succession. Average *per capita* (a and b) and population (c and d) effect of *Enoplochiton niger*, on mean (a and c) and variance (considered as ‘effect size’) (b and d) of percent cover of bare rock, and ephemeral and crustose algae at the study site in Punta Talca. Bars are 95% confidence intervals estimated through a bootstrapping procedure. Inserts in b and d correspond to *per capita* effects on mean and variance cover, respectively, of *E*. *niger* on *D*. *antarctica* plantlets fronds growth and total biomass evaluated with plantlets transplant in the field experiments 2. Hatched bars in b) correspond to the collective effect of grazers measured in control treatment on variance of algae.

Estimation of *per capita* effects of *E*. *niger* showed positive and significant effects on bare space cover throughout the study, which agrees with the negative impacts evidenced for on ephemeral algae (Fig 4A). No significant *per capita* effects were observed on crustose algae (i.e. confidence intervals crossing zero, Fig 4A). In the case of *L*. *spicata*, *E*. *niger* completely precluded its colonization within enclosures compared with exclusion areas (see above). Similar effects were observed for control areas, where the collective effect of grazers impeded *L*. *spicata* settlement. Thus, we could not estimate *per capita* effects on this alga. In the other scenario, because bull kelp abundance at the site was negligible during experiment 1 (effects on colonization), we estimated interaction strength between chitons and bull kelps considering frond growth and biomass of small plantlets transplanted inside experimental areas during the second series of experiment (see insert in Fig 4A). In agreement with effects on total frond length and biomass, we found that *per capita* effects of *E*. *niger* on plantlet frond growth was not different from zero (see black circles, insert in Fig 4A). Similarly, the *per capita* effect of chitons on plantlet biomass was not significant (i.e. confidence intervals crossing zero, insert in Fig 4A).

Averaged effects of *E*. *niger* on spatial variability of bare rock was positive and significant (bars not crossing zero), but not significant neither on ephemeral nor on crustose algae (Fig 4B). *E*. *niger* average effects on spatial variation of *D*. *antarctica* plantlets growth and biomass also were negligible and not different from zero (see insert Fig 4B).

Population-level estimations of effects considered natural herbivore densities at the study site and those estimated at different sites across the range overlap (Fig 4C and 4D). Estimation of population effects of *E*. *niger* showed that they were able to produce and maintain (during the algal colonization phase) around 90% of bare rock cover per m^2^ per day in the study site (black circles in Fig 4C). This is concordant with the strong negative effect expected for this grazer on opportunistic/ephemeral algae, since at natural densities they might remove around 46% cover per m^2^ per day of this functional group (Fig 4C). Averaged collective effects of all grazers found in control areas were lower than expected for local population effects of *E*. *niger* for bare rock (hashed bars, Fig 4C). The effect of all grazer species on plantlets of *D*. *antarctica* in control areas, was not significantly different from zero for both growth and biomass (see white squares, insert in Fig 4A).

Expected average population effects of *E*. *niger* on spatial variation of bare rock showed that natural population of this grazer could significantly increase space heterogeneity, while non-significant effects on spatial variance of ephemeral or crustose algae were observed (hashed bars, Fig 4D). The average collective effect on spatial variation of bare rock for control areas was low, suggesting high densities of *E*. *niger* and/or that the effects of multiple grazers were not additive (Fig 4D).

**Fig 5:**
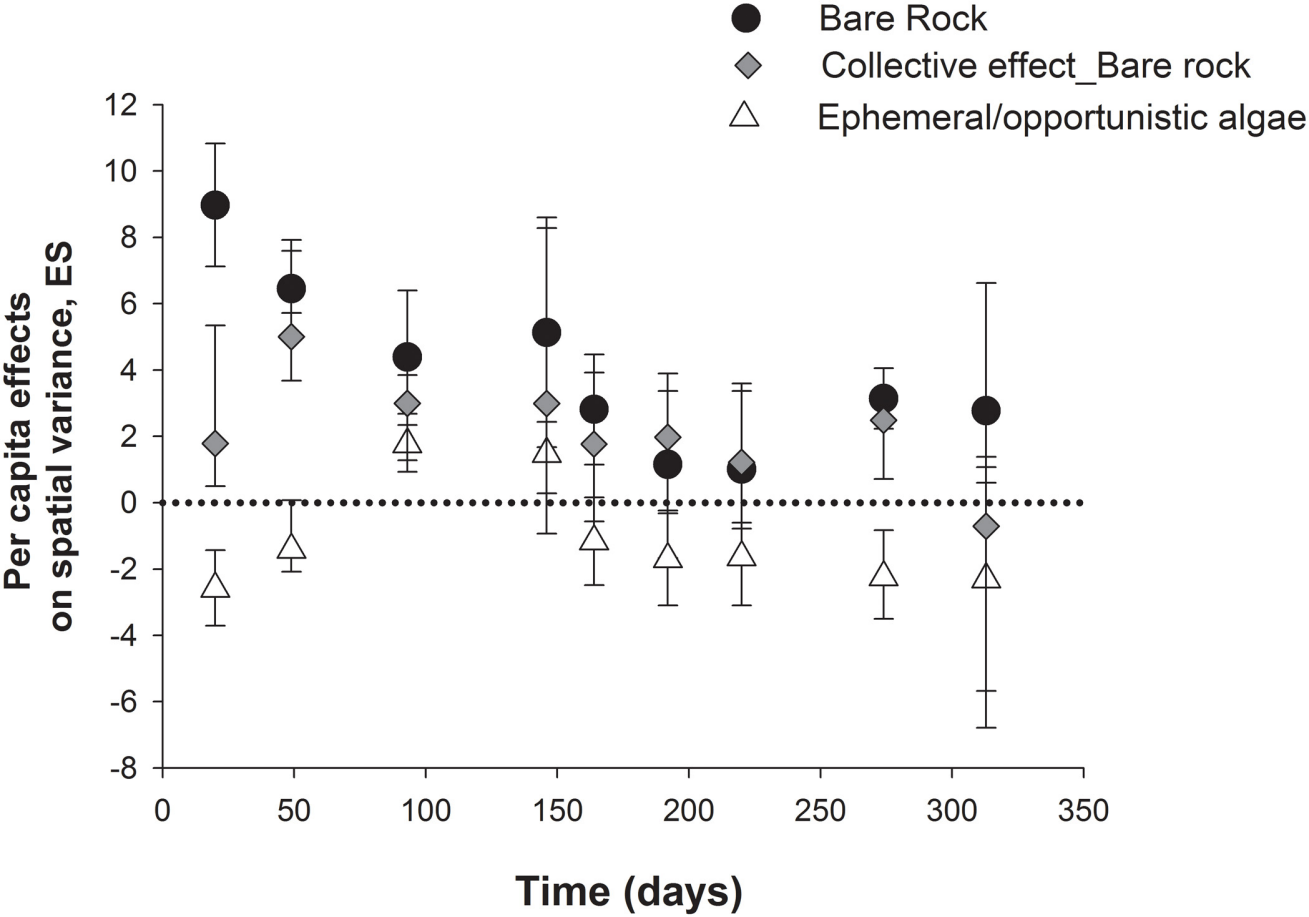
Temporal variation of the interaction between the grazer *E*. *niger* and algae and bare rock production recorded in the field experiments. *Per capita* effects of *E*. *niger* on spatial variance of bare rock, ephemeral and crustose algae recorded at different times of the experiment 1 (see text for details). Collective effects of all herbviores present in the study sites (recorded in open areas) are also presented (crossed black diamonds). Bars are 95% confidence intervals estimated through a bootstrapping procedure.

Analyses of temporal trends in spatial variation of the different algae group and bare rock, showed that increase of spatial variance of bare rock by *E*. *niger* was persistent throughout the experiments (black dots, Fig 5), similar to the collective effects recorded in control areas (crossed diamonds, Fig 5). Effects on opportunistic/ephemeral algae showed an increase of its spatial variance at early stages of the experiment coincident with early succession and a posterior decrease of spatial variance at intermediate stages (white triangle, Fig 5). Crustose algae spatial variation influenced by the grazer showed a non-significant (i.e. points crossing zero) effect during early stages followed by a slight increase of spatial variance at the intermediate times of the experiment (gray squares, Fig 5).

**Fig 6:**
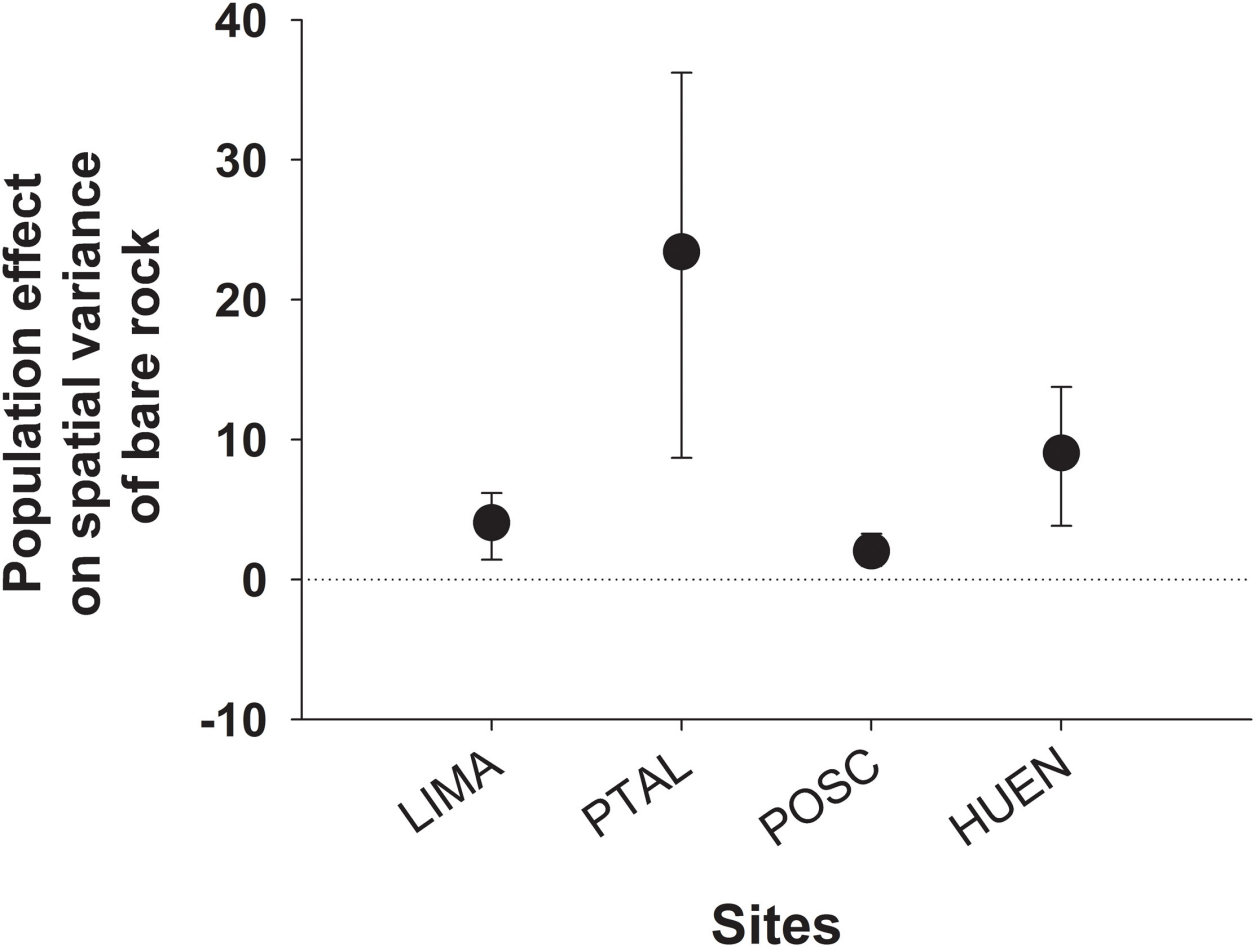
Expected population-level effect of *E*. *niger* on spatial variation (effect size) of bare space distribution across different sites where the grazer and algae species overlap. Local densities of *E*. *niger* and *per capita* effects recorded in field experiments in Punta Talca were used to calculate populational effects (see text for details).

Considering bare rock production/maintenance inside experimental enclosure plots as a proxy for a strong grazing/bulldozing effect, we evaluated the expected averaged populational effect of *E*. *niger* across sites where coexist with the brown algae species. Given large densities of *E*. *niger* observed across the range overlap with *L*. *spicata* and *D*. *antarctica*, a strong averaged populational effect on bare rock production is expected across this region, (i.e. 38% (± 0.5) per m^2^ per day, Fig 6), which are expected to be highly variable across the overlap region (coefficient of variation = 0.92, and see Fig 6).

## Discussion

Our results show that *E*. *niger* can reach high population densities and large body size, which are reflected in strong effects on algal colonization, abundance, and spatial distribution, even in a location at its geographic range limit. Grazing/bulldozing effects during early and mid-succession precluded settlement of most algae, generated spatial structure through the opening primary space, and homogenized the distribution of opportunistic/ephemeral algae at small spatial scales. Grazing effects on dominant algal species were variable through increasing long-term spatial heterogeneity in the study system. This patch-generating mechanism is compounded by the variable population-level effects of *E*. *niger* across its range edge, which are expected to follow the differences in densities observed across sites. The controlling effects of grazers on the settlement of large brown algae seemed to be concentrated on very early life stages, suggesting that most large brown algae reach an escape size early in their ontogeny. In agreement to our results, previous stomach-content analyses indicate that *E*. *niger* can have a key role in the structuring of benthic communities, as is able to assimilates a broad spectrum of prey, ranging from macroalgal spores to small invertebrates [58]. Strong and variable grazing by *E*. *niger* can have important consequences for the spatial configuration of kelp-dominated communities in northern Chile. Here, we discuss the key roles of the intertidal grazer in setting community composition, recovery, and spatial structure in low-shore habitats across its range edge.

### Strength and variability of *E*. *niger* grazing effects on spatial heterogeneity of the algal assemblage

It has been suggested that functional traits like consumers’ density, foraging behaviour, and plant phenology can be relevant to predict consequences of herbivore-alga interactions at different spatial scales [10,12,71]. For example, distribution of grazers during foraging can sometimes be a good predictor of resource distributions at small spatial scales [11,22]. We found that spatial distribution of *E*. *niger* individuals while foraging was random during summer and winter in our study site, similar to which has been found for other chitons (e.g. *Chiton granosus* [72]). Probably, random foraging determines uniform or random bare rock distribution as we observed in open plots in field experiments, thus increasing among-sites spatial variance. Despite large population densities of this grazer were observed in the study site, random distribution at foraging suggests this species does not form “feeding fronts” as observed for other abundant grazers (e.g. sea urchins, littorinids snails, see [73] for review). Given the large body size of *E*. *niger* relative to other molluscs of the assemblage [49], we would expect large distances at foraging and long foraging times [74,75], which might account for the large *per capita* effect of *E*. *niger* observed in our study—this finding deserves future attention.

One of the most important question for the management of different ecosystems is when does grazing increase the spatial heterogeneity of vegetation? [11]. In general, it is expected that consumers with strong but with variable effects increase resource heterogeneity with low residual variation (e.g. due to physical factors) [25]. In our experiments, we observed large and temporally variable effects of *E*. *niger* on bare rock production and the abundance of opportunistic/ephemerals algae. The temporal variability in consumer effects seems to be the consequence of successional changes in algal colonization, the seasonal environmental variability characteristic of northern-central Chile, or an interaction of both processes [76–78]. In general, it is expected that the effects of grazing on spatial heterogeneity of vegetation depend on the spatial scales of grazing and vegetation distribution [79]. Thus, homogeneous grazing often occurs at smaller spatial scales, where randomly distributed grazing overrides fine-scale spatial heterogeneity in vegetation created by environmental heterogeneity or neighborhood interactions [11]. Grazing by *E*. *niger* individuals generated patches of bare rock of size variable inside enclosure plots (i.e. from 2 to 10 cm^2^ approximately) which translated in large and constant “among-plots” variance through time. Concurrently, cover of opportunistic/ephemeral algae within *E*. *niger* enclosures was low and mostly variable through time compared with grazer exclusion areas, in which these algae maintain large cover, low variability, and thus high spatial homogeneity. Notwithstanding, effect of *E*. *niger* on spatial variance of opportunistic algae was also variable through the course of the experiment. This suggests that inherent variation of colonization rates of these algae through succession might change the direction and magnitude of grazer effects on algae distribution at small scales.

Given the joint effects of functionally similar benthic grazers, such as chitons and small limpets, a strong control of these species on opportunistic/ephemeral algae is expected, with a smaller impact on adult, established, algae (see below). We also observed that the collective effects of grazers in control areas (open plots) were lower than those expected for *E*. *niger* population in the study site, suggesting that at larger densities, within-guild competition (e.g. interference) could be relevant to dampen the effects of this grazer. Thus, strong small-scale collective effects and interspecific constraints of the guild could contribute to maintain large-scale spatial heterogeneity in this system. Likely, population self-regulation can preclude the potential for a phase-shift of the system into the barren grounds, as observed for other grazers [80]. However, given the large body size and densities of *E*. *niger* (maximum body length = 10±0.13 cm [49]), which translates in large *per capita* and population level effects, and that large keyhole limpets are removed by humans [63],limited redundancy [19] in effect is more probable in this system. Judging by the effects observed in *C*. *granosus* [62], which is also abundant in low shore habitats and reaches medium-size, this species could have equivalent maintenance/production of bare space to *E*. *niger* but only at larger population densities. Given that bare space is a limiting resource for settlement of new individuals for most intertidal species [81], it is critical the role that *E*. *niger* plays maintaining the dynamic mosaic of the low intertidal landscape. Further studies are needed to determine if the absence of *E*. *niger* beyond its southern range edge (~31°S) translates to relevant changes in the structure of the low shore intertidal community, or if other functionally similar or equivalent grazer could compensate for its absence.

### Direct and indirect grazing/bulldozing effects on large brown algae structure

Regarding the patchy distribution and low density of the bull kelp *D*. *antarctica* present in the study platforms, edge populations of this alga could be demographically unstable and probably prone to local extinction, as shown for other taxa elsewhere [82,83]. This suggestion agrees with recent phylogeographic studies suggesting that populations of the bull kelp at their northern edge may represent recent re-colonization of a marginal habitat [84]. Therefore, intense grazing by *E*. *niger* could be also critical at this range influencing persistence of potential ‘satellite’ populations of this alga together with abiotic factors operating as a barrier to dispersal and settlement [85,86]. Thus, this herbivore-kelp interaction could be considered a useful model system to explore the role of consumer species into influencing geographic limits of algae. Further studies are needed (via transplant experiments) to determine if grazing can potentially constrain their ability to colonize sites northern the range edge.

We found positive and significant spatial correlations between *E*. *niger* and adult *L*. *spicata*. *Lessonia spicata* (as well as *L*. *berteroana* in sites northern the range overlap) can act as a shelter for different invertebrate species, constituting a “habitat-forming species” [39,54]. In our study sites, *E*. *niger* individuals commonly rested underneath *L*. *spicata* canopies that could serve as shelter from desiccation stress during low tide.

The early stages of large brown leathery algae are highly vulnerable to grazing (see [10] for review). Given high abundances of *E*. *niger* recorded at the study site and across the range overlap, local populations of this grazer are expected to have strong negative effects on *L*. *spicata* recruitment. In line with this, we observed that *L*. *spicata* exclusively settle inside grazer-exclusion areas, but not in *E*. *niger* enclosure nor in open areas (controls) where grazing was most intense (in enclosures and open areas, bare rock covered ca. 85% of the substrate through the span of the experiment). Similarly, absence of bull kelp recruits inside enclosures and open access areas during the first series of experiments suggests that natural populations of *E*. *niger*, together with other functionally similar and abundant herbivores like *C*. *granosus*, may limit the abundance of *D*. *antarctica* through removal of propagules. Notwithstanding, the absence of bull kelp recruits from grazer-exclusion plots in our first series of experiments may have been caused by the small population sizes present in the study site and pre-emptive competition by opportunistic green algae [87,88] and adults of *L*. *spicata* [39,54]. Indeed, we observed a negative and significant spatial correlation of *D*. *antarctica* recruits and adult *L*. *spicata* in our study site, suggesting bull kelp recruitment could be affected by, for example, the mechanical effect of *L*. *spicata* fronds (“whiplash effect” see [54]). The low effects of *E*. *niger* on *D*. *antarctica* growth and biomass, however, suggest that when plantlets are able to establish in the substrate and reach a certain size, they are likely less palatable or vulnerable to *E*. *niger* or other grazers. We had no evidence of fish grazing on plantlets, which are critical factors determining *D*. *antarctica* distribution and abundance in other systems [61]. Patches of coralline algae that provide shelter against grazing [35,53] could enhance the recruitment and chances for survival of kelps (*Lessonia* spp.). In our field experiments we observed small patches of coralline crusts in both exclusion and enclosure plots, but *L*. *spicata* settled only in exclusion areas. This spatial pattern corresponds well with the clumping at small scales (i.e. 50–100 cm) found for both juvenile and adult *L*. *spicata* and for crustose algae. Similar patchy distribution at intense to moderate grazing regimes has been reported recently for the sister species, *L*. *berteroana*, in more protected sites in northern Chile [35]. The clumped pattern of recruits found for *L*. *berteroana* has proved to be important for coalescence, which can increase survival potential [35]. Given that chitons are expected to graze efficiently on coralline algae crusts [59], it is probably that intense grazing by *E*. *niger* upon coralline crusts observed in our experiments precludes *L*. *spicata* recruitment. Nonetheless, coralline algae may well constitute shelter against other grazers (e.g. fissurellid limpets) that cannot forage upon the strong structure of coralline crusts [59,60]. Thus, in our system, spatial heterogeneity (e.g. shelter availability, see [87]) can play a role influencing the ability of both *L*. *spicata* and the bull kelp spores to survive in presence of strong grazing of spores and germlings by *E*. *niger*. Large brown algae are intensely harvested in Chile and support a lucrative economic activity [35,45]. According to our results, the concomitant alterations of grazer abundances, algal re-colonization, and the spatial structure of the landscape after the loss of kelps should be considered for appropriate ecosystem management.

## Conclusion

Our results suggest that the large chiton *E*. *niger* has a key ecological role as modifier of the spatial structure of the kelp-dominated low intertidal community in northern-central Chile. It plays this role by regulating dominant algal species and the spatial distribution and abundance of open space. According to our main results, this grazer could be considered a strong modifier of the intertidal landscape. Considering the importance of large brown algae species in the economy of “algal-harvesters” in northern Chile, and that *E*. *niger* is not harvested in these communities compared with other grazers (e.g. fissurellid limpets and fish), knowledge on the spatial and temporal variation of population densities, size structure, and foraging patterns of this chiton across its geographic distribution seems to be a relevant factor to consider for both conservation and management strategies.

## Supporting Information

S1 File Details of field-based experimental design and setup, and tables with statistical results. Exclusion/enclosure method and effectiveness: Preliminary experiments, experimental design and procedures (**Figure A**). Repeated measures ANOVA of a) bare rock and b) ephemeral algae (i.e. *Ulva compressa*, *U*. *rigida*, *Pyropia sp*.) found inside the experimental plots of the field experiment (**Table A**). Split-plot analysis of variance of *D*. *antarctica* plantlets fronds length a), and wet weight b) transplanted into acrylic plates in different grazer treatments in the field experiment 2 (**Table B**).
(PDF)
