# Supplementary material for: Herbivore-Alga Interaction Strength Influences Spatial Heterogeneity in a Kelp-Dominated Intertidal Community
Source: PLoS One. 2015 Sep 11;10(9):e0137287. doi: 10.1371/journal.pone.0137287 (PMC4567380; doi:10.1371/journal.pone.0137287)
Supplement: S1 File — Exclusion/enclosure method and effectiveness: Preliminary experiments, experimental design and procedures (Figure A). Repeated measures ANOVA of a) bare rock and b) ephemeral algae (i.e. Ulva compressa, U. rigida, Pyropia sp.) found inside the experimental plots of the field experiment (Table A). Split-plot analysis of variance of D. antarctica plantlets fronds length a), and wet weight b) transplanted into acrylic plates in different grazer treatments in the field experiment 2 (Table B). (PDF) [file pone.0137287.s001.pdf]

Supporting information S1:
Details of field-based experimental design and setup
and
Tables with statistical results

Herbivore-algae interaction strength influences spatial heterogeneity in a kelp-dominated intertidal community

Moisés A. Aguilera[1*], Nelson Valdivia[2] and Bernardo R. Broitman[1]

[1]Centro de Estudios Avanzados en Zonas Áridas (CEAZA), Universidad Católica del Norte, Larrondo 1281, Coquimbo, Chile.

[2]Instituto de Ciencias Marinas y Limnológicas, Facultad de Ciencias, Universidad Austral de Chile, Campus Isla Teja s/n, Valdivia, Chile

Details of field-based experimental design and setup

Exclusion/enclosure method and effectiveness: Preliminary experiments

In September 2009, we conducted a preliminary study to examine the effectiveness of using stainless steel fences to exclude, and enclose in, *Enoplochiton niger* from experimental areas; we additionally evaluated the potential of the fencing method to resist wave action in the study site at Punta Talca. We selected nine 35 × 35 cm areas of rocky substratum (about 40% cover of bare rock and 50% coralline algae). Each of these plots was randomly assigned to one of the following three treatments (Fig. S1): (a) total grazer exclusion (complete fence), (b) one-chiton enclosure (complete fence), in which one adult individual of *E. niger* (mean ± Standard Error of the Mean of maximum body length: 9.3 ± 0.11 cm) was enclosed and (c) procedural control (partial fence) where only the corners of the experimental plots were fenced (20 cm of fence in each corner). Thus, 20 cm on each side were fenced, while the remaining 15 cm in the centre were left open to allow immigration of benthic invertebrates. All these areas were scraped clean with metallic brushes. We used three replicates of each treatment. Fences were 7 cm high and 7 mm mesh opening, and were fastened to the rock with stainless-steel bolts. The experiment was maintained for 30 days in the field and checked every two weeks to assess fence condition (damaged, removed) and effectiveness to exclude both grazers and predators from treatment (a), enclose the grazer in treatment (b), and to assess other non-anticipated effects of fencing on the recolonization of the experimental plots (c). We took photographs using a digital camera positioned directly above each plot and in adjacent 35× 35cm "open areas" around them (n=9) which were also cleaned but not fenced at the start of the experiment. Each photograph was cropped to include only the quadrat and analysed using the image J software (http://imagej.- nih.gov/ij/download.html). Percentage cover of algae colonizing each plot was quantified by projecting 25 dots randomly onto each photo and assigning a value of 4% to each organism that occurred in each dot.

In general, complete fences were effective to reduce/impede grazer entry inside plots and to maintain all chitons inside throughout the pilot study. Only one fence was damaged in the course of the study with sides pried by waves and kelp whiplash. Most partial fences (procedural control) were easily damaged by waves in all plots, and mechanical effects of dislodged pieces of steel were evident on the substrate inside plots through marks on the bare rock. At the end of the field assays (30 days) total grazer exclusion had 35.7% (± 5.77) mean cover of green ephemeral algae (mostly ulvoids) compared with 30% (± 14.4) mean cover observed in one-chiton enclosure. After partial fences were dislodged, we found no herbivores inside procedural controls compared with adjacent bare areas (no-fenced). We observed only 14% (± 2.0) mean cover of green ephemeral algae in procedural control with 70.3% (± 3.52) of bare rock mean cover present there. Open areas (no-fenced) around the nine fenced plots, showed 6.56% (±2.53) mean cover of ephemeral algae with 82.4 %(±6.81) of bare rock mean cover. The differences in ephemeral algae cover between no-fenced areas and partial fenced ones was 0.77% for bare rock and 12.1% for ephemeral algae. Thus our study showed that using stainless steel fences was an effective experimental procedure to control benthic grazer abundance in the low-shore habitat. Additionally, our preliminary observations suggest that considering partial fences as a procedural control can cause undesirable "artefacts" in the experimental design as they are easily removed by waves potentially affecting the rates of spore colonization and thus increasing bare rock cover. It is worth noting that a partial barrier as an

enclosure/exclusion procedural control has been considered inadequate for estimating artefacts associated with exclusion treatments [1,2]. Despite "cageless" methods to exclude benthic grazers as copper paint have been used as alternative to fencing [3,4], these methods are only partially effective to exclude other grazers present in the intertidal assemblage (e. g. keyhole limpets [5,6]) compared with fencing the experimental plots.

Experimental design and procedures: Experiment 1

In the first experiment, we considered (a) *E. niger* enclosures, (b) benthic grazer exclusion and (c) control (open areas) treatment (Fig A). We used the enclosure/exclusion method described and validated in the previous section (Preliminary experiments) to set up these experimental treatments. Plots were reset to the initial successional stage of bare rock by scraping the rock surface clean with drill-mounted brushes and manual chisels. This procedure allowed us to remove all organisms including encrusting algal fragments. Since *E. niger* is the main grazer species at low- intertidal zones on these shores, we assumed that experimental grazer enclosures resembled natural conditions of grazing pressure. However, other grazers also occur in these shores, which can generate differences between the enclosure and control (open areas) treatments (Fig A). Given that partial fences are easily removed by waves and can cause undesirable effects on algae colonization, this procedural control treatment was not further considered in the final experimental design. Because we did not include a "fence effect" treatment in this experiment (Fig. Ad), we were unable to estimate if a fence-only treatment increased algal cover (ephemeral algae) in exclusion areas compared with the 'open access' areas (Fig. Ac). Notwithstanding, preliminary study showed that percentage cover of bare rock was only 12.1% higher in adjacent open areas compared with partially fenced plot with only 0.77% less ephemeral algae in the former compared with the last. In this point, as we focus on the spatial effects of *E. niger* enclosed inside fences, considering total exclusion areas as the 'referential condition' (true control), which had the same conditions as enclosures, we expected to capture the general patterns of grazing effects on spatial heterogeneity. Additionally, previous studies suggest that different exclusion methods (e.g. plastic brush and copper paint) do not seem to alter significantly algal recolonization in rocky intertidal habitats in the study region when compared with open areas. Also, most effects in these experimental arenas are attributed to the grazing of the diverse intertidal herbivore assemblage [4,6]. Ephemeral algal cover found in exclusion plots, grazer enclosures and control (see Results section in the Main text) were within the range reported for the same treatments considered in other studies [4,5]. Given these information, we expected that the effect of fences, if any, on altering algal cover to be minimal.

We monthly removed other grazers found inside enclosure/exclusion plots in the experiments such as *Scurria* spp. recruits (2-3 individual per plot >5mm length). In general, the number of other grazers, e.g. *Scurria* spp., removed per month from plots was low (<2).

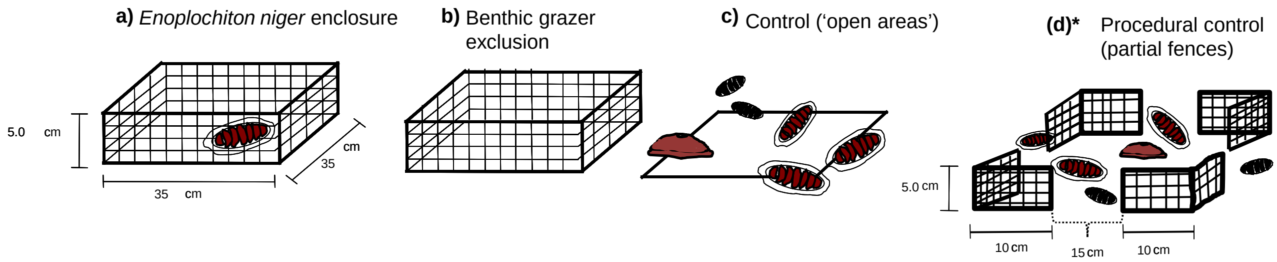

Figure A. Experimental design of field experiments utilized to test the effect of herbivores on the ability of algae to colonize and persist in the low intertidal community at Punta Talca. Rock was scraped clean at the beginning of the experiments and algae allowed to colonize and grow. *As previous field assays showed that considering 'partial fences' as Procedural control (d) can add an additional artefact to our experiments, this treatment was not further considered in the grazer enclosure/exclusion experiment.

Cited Literature

1. Johnson LE. Potential and peril of field experimentation: The use of copper to manipulate molluscan herbivores. J Exp Mar Bio Ecol. 1992;160: 251–262. doi:10.1016/0022-0981(92)90241-2

2. Benedetti-Cecchi L, Cinelli F. Confounding in field experiments: direct and indirect effects of artifacts due to the manipulation of limpets and macroalgae. J Exp Mar Bio Ecol. 1997;209: 171–184.

3. Range P, Chapman MG, Underwood AJ. Field experiments with "cageless" methods to manipulate grazing gastropods on intertidal rocky shores. J Exp Mar Bio Ecol. 2008;365: 23–30. doi:10.1016/j.jembe.2008.07.031

4. Aguilera MA, Navarrete SA. Effects of Chiton granosus (Frembly, 1827) and other molluscan grazers on algal succession in wave exposed mid-intertidal rocky shores of central Chile. J Exp Mar Bio Ecol. 2007;349: 84–98. doi:10.1016/j.jembe.2007.05.002

5. Aguilera MA, Navarrete SA. Functional identity and functional structure change through succession in a rocky intertidal marine herbivore assemblage. Ecology. 2012;93: 75–89. doi:10.1890/11-0434.1

6. Nielsen KJ, Navarrete SA. Mesoscale regulation comes from the bottom-up: intertidal interactions between consumers and upwelling. Ecol Lett. 2004;7: 31–41. doi:10.1046/j.1461-0248.2003.00542.x

Table A. **Differences in available space and algae among treatments.** Repeated measures ANOVA of a) bare rock and b) ulvoids (i.e. *Ulva compressa*, *U. rigida*) found inside the experimental plots of the *Enoplochiton*-*Durvillaea* field experiment. Since planned contrasts were not orthogonal, P-values were adjusted using Dunn-Sidák correction. P<0.05*, p<0.01**.

Source	df	MS	F	P
**a) Bare rock**				
Between Subject				
Treatment	2	22.284	110.4	<0.0001**
Error	9	0.2018		
Planned Constrasts				
Control vs. Enclosure	1	2.0679	10.25	0.0321*
Control vs. Exclusion	1	40.512	200.77	<0.0001**
Enclsoure vs. Exclusion	1	24.274	120.3	<0.0001**
Within Subjects				
Time	8	0.2979	2.63	0.0199*
Time*Treatment	16	0.1454	1.29	0.2439
Error (Time)	72	0.1132		
**b) Ulvoids**				
Between Subject				
Treatment	2	14.401	22.94	0.0003**
Error	9	0.6277		
Planned Constrasts				
Control vs. Enclosure	1	4.392	7.00	0.0780
Control vs. Exclusion	1	28.372	45.20	<0.0001**
Enclsoure vs. Exclusion	1	10.438	16.63	0.0084**
Within Subjects				
Time	8	0.1526	1.04	0.4078
Time*Treatment	16	0.2005	1.37	0.2138
Error (Time)	72	0.1464		

Table B. **Grazing on *D. antarctica* plantlets.** Split-plot analysis of variance of *D. antarctica* plantlets a) fronds length, and b) wet weight transplanted onto acrylic plates in different grazer treatments in the field experiment 2 (see text for details); **ns**: non-significant values (α=0.05). Position corresponds to the place where plantlets were glued upon acrylic plates in each experimental plot. The error term to test the significance of the model was the replicate plots nested in each treatment. Analyses were conducted on average change of plantlets length and wet weight after three repetition of the transplant procedure. Treatments design consisted in: a) *E. niger* enclosure, b) Grazers exclusion, c) Control (open areas), and c) procedural control (partial fences), see main text for details.

**a) Length**					
SV	df	SS	MS	F	P
Treatment (T)	3	0.01391	0.004637	0.479	0.706^{ns}
Position (P)	1	0.01988	0.01988	2.172	0.179^{ns}
P*T	3	0.02975	0.009917	1.084	0.410^{ns}
Residuals	8	0.07322	0.009152		
**b) Weight**					
Treatment (T)	3	0.000655	0.0002185	0.212	0.885^{ns}
Position (P)	1	0.000261	0.000261	0.212	0.658^{ns}
P*T	3	0.005281	0.0017605	1.426	0.305^{ns}
Residuals	8	0.009874	0.0012343		
